# Supplementary material for: Inner Retinal Layer Changes Reflect Changes in Ambulation Score in Patients with Primary Progressive Multiple Sclerosis
Source: Int J Mol Sci. 2023 Aug 17;24(16):12872. doi: 10.3390/ijms241612872 (PMC10454007; doi:10.3390/ijms241612872)
Supplement: Supplementary file 1 [file ijms-24-12872-s001.zip › ijms-2542765-supplementary.pdf]

International Journal of
Molecular Sciences

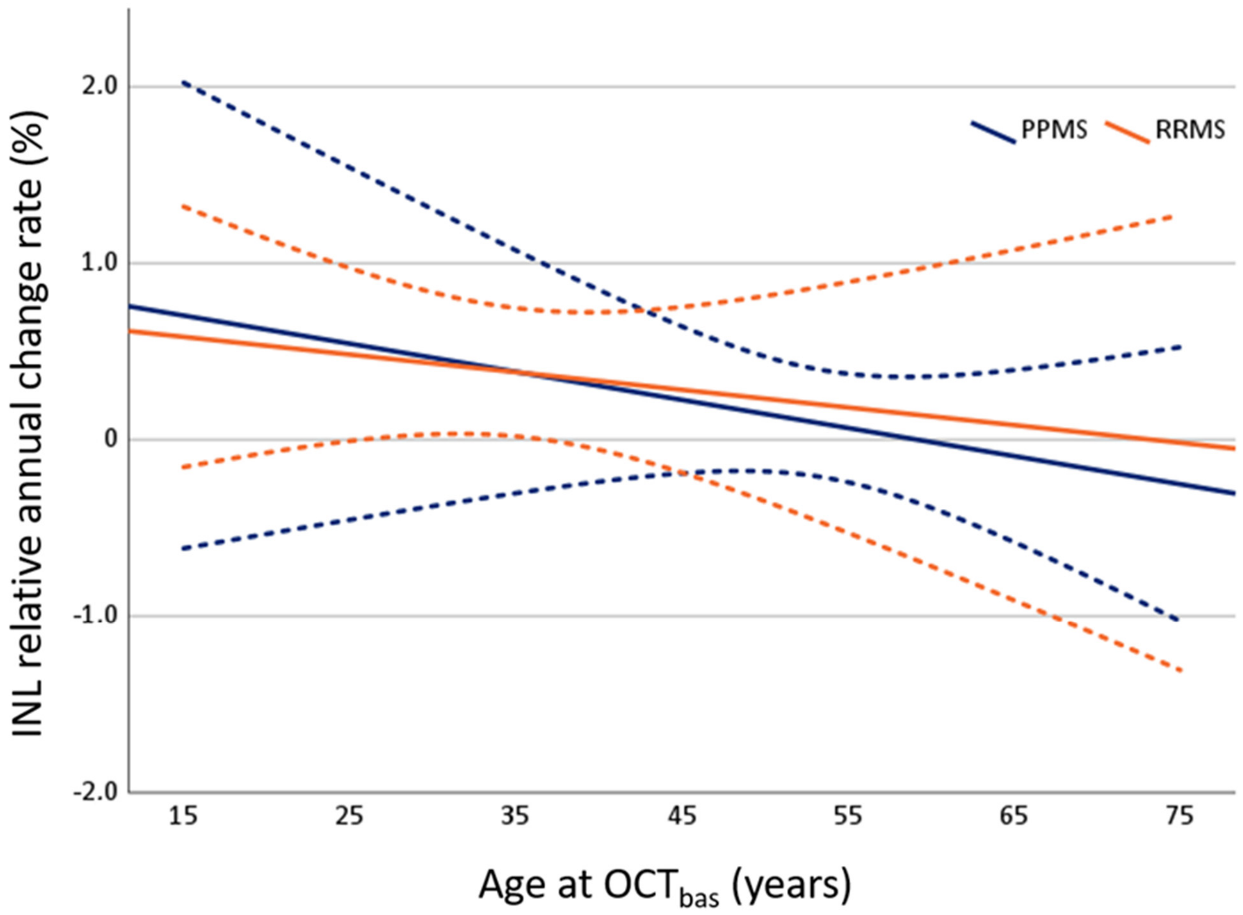

Figure S1: Relative annual change rate of INL in patients with PPMS and RRMS.

Mean relative annual change rate of the macular inner nuclear layer (INL) (in %) is shown for persons with multiple sclerosis (PwMS), either with a relapsing-remitting (RRMS, orange; n = 72 eyes) or a primary progressive course (PPMS, blue; n=61 eyes) in relation to age at baseline OCT (OCT_{bas}) and after exclusion of extreme lower and upper outliers according to Tukey (1st quartile-3xIQR<range included<1st quartile+3xQR). The 95%-confidence intervals (dotted lines) point out to the difference in age at baseline OCT for the different MS subtypes.
